# Supplementary material for: Accounting for long-range correlations in genome-wide simulations of large cohorts
Source: PLoS Genet. 2020 May 5;16(5):e1008619. doi: 10.1371/journal.pgen.1008619 (PMC7266353; doi:10.1371/journal.pgen.1008619)
Supplement: S1 Appendix — (PDF) [file pgen.1008619.s001.pdf]

S1 Appendix. Wright-Fisher Implementation Details

We describe here the precise order of events happening at each generation in our implementation of the Wright-Fisher model. For more technical details see the documentation at `https://msprime.readthedocs.io`, as well as the source code at `https://github.com/tskit-dev/msprime`.

In the first ('current') generation, samples are initialized as haploid copies of the region to be simulated (which can later be paired to form diploid individuals). Lineages of each sample are then constructed backwards in time as follows (detailed comments labelled by pseudocode line number are provided below):

Algorithm 1 Wright-Fisher simulations in `msprime`

```
1: time ← 0
2: while number of extant lineages > 0 do
3:     time ← time + 1
4:     migrate lineages (migration rates, time)
5:     apply demographic events (time)
6:     choose parents for all extant lineages
7:     recombine extant lineages
8:     record coalescence events
```

4 Migration events are drawn according to the forwards-time rates provided, and migrant lineages are moved to their new population. This is equivalent to migration of gametes, as opposed to migration of diploid individuals. A forwards-time event from population i to j moves a lineage from population j to i backwards in time.

5 Demographic events are carried out, such as changes to population sizes or growth rates, mass migrations, or bottlenecks.

6 Each haploid lineage draws a diploid parent within its current population.

7 Recombination occurs, with each breakpoint alternately assigning segments to be inherited from one of the two parental copies of the genome (back-and-forth recombination, see Fig 1 in the main text).

8 Segments inheriting from the same parental copy of the genome are merged into a single lineage, with coalescent events recorded in overlapping regions.

When there is a single ancestral lineage at every position in the simulated genome, the simulation terminates.

Our whole-genome simulations are performed with a single chromosome of length 35.13 Morgans and 22 ‘effective’ chromosomes of realistic lengths separated by 0.5 Morgans. This is not exactly equivalent to simulating fully independent chromosomes. However, this should not have a qualitative impact on the analyses considered here.
