# Supplementary material for: Accounting for long-range correlations in genome-wide simulations of large cohorts
Source: PLoS Genet. 2020 May 5;16(5):e1008619. doi: 10.1371/journal.pgen.1008619 (PMC7266353; doi:10.1371/journal.pgen.1008619)
Supplement: S2 Appendix — (PDF) [file pgen.1008619.s002.pdf]

S2 Appendix. Long-range linkage disequilibrium

For pairs of loci at low recombination distances ($r \ll 1$), it is unlikely for more than a single recombination event to occur in a given meiosis. In this case, the coalescent accurately models LD between linked loci. For larger recombination distances, loci only become unlinked under an odd number of recombinations. This has probability $P(\text{odd \# rec. events}|r) = \frac{1-e^{-2r}}{2}$, which has a maximum value of 1/2. This leads to non-zero long-range LD, even in the case of fully unlinked loci [1]. The diploid Wright-Fisher captures this, but coalescent estimates of LD decay to zero for increasing r (Fig A).

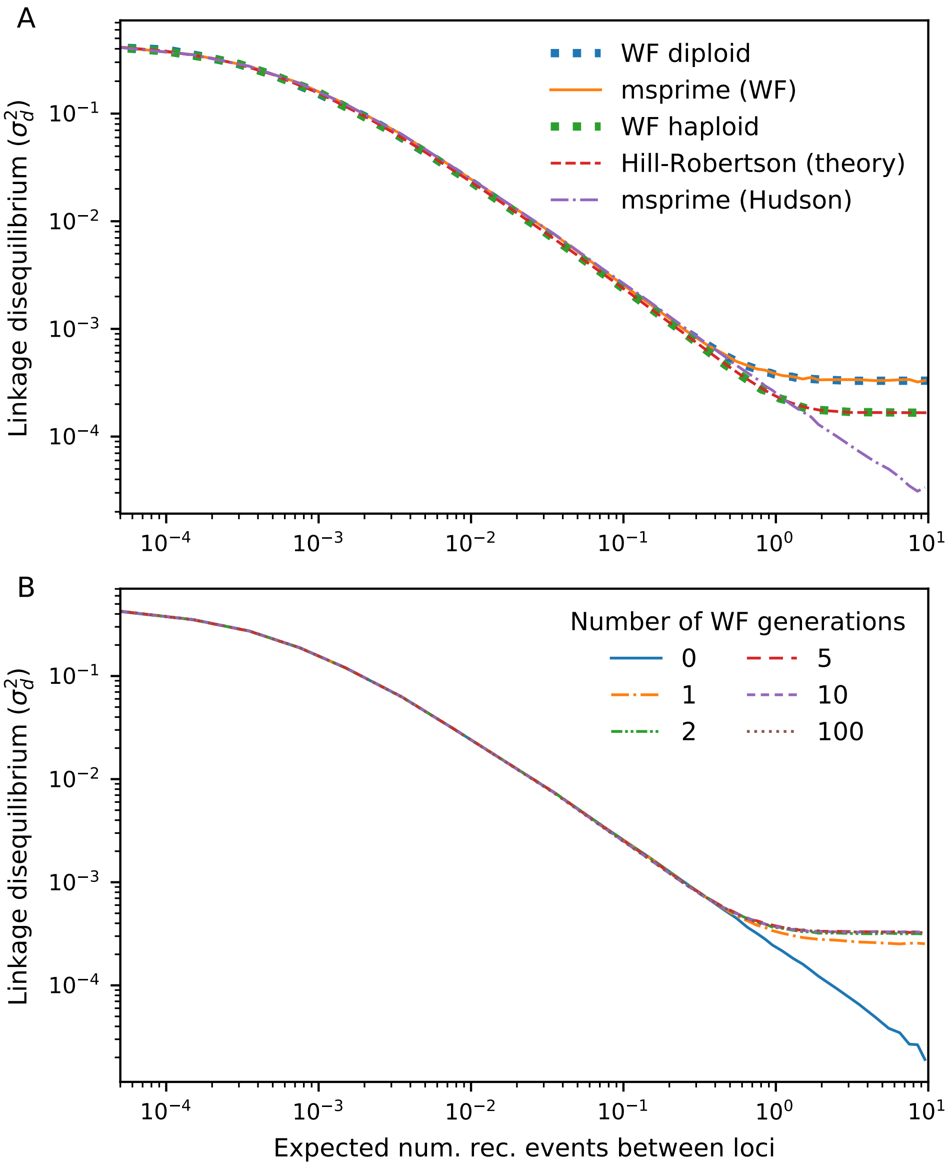

Fig A. Linkage disequilibrium as measured by $\sigma_D^2 = E[D^2]/E[p(1-p)q(1-q)]$ under different simulation and theory models [2]. Simulations were carried out with population size $N = 1000$ at steady state demography for a single 10M chromosome. At fully unlinked loci, the expected value of σ_D^2 is $\frac{1}{3N}$ in a diploid model and $\frac{1}{6N}$ in a haploid model [3]. (A) Hudson and Wright-Fisher simulations. (B) Hybrid simulations with varying numbers of Wright-Fisher generations before switching to the Hudson coalescent.

References

[1] L. King, J. Wakeley, and S. Carmi. "A non-zero variance of Tajima's estimator for two sequences even for infinitely many unlinked loci". In: *Theoretical Population Biology* 122 (2018), pp. 22–29.

[2] W. G. Hill and A. Robertson. "Linkage disequilibrium in finite populations". In: *Theoretical and Applied Genetics* 38.6 (1968), pp. 226–231.

[3] A. P. Ragsdale and S. Gravel. "Unbiased Estimation of Linkage Disequilibrium from Unphased Data". In: *Molecular Biology and Evolution* (Nov. 2019).
