# Supplementary material for: Accounting for long-range correlations in genome-wide simulations of large cohorts
Source: PLoS Genet. 2020 May 5;16(5):e1008619. doi: 10.1371/journal.pgen.1008619 (PMC7266353; doi:10.1371/journal.pgen.1008619)
Supplement: S3 Appendix — (PDF) [file pgen.1008619.s003.pdf]

S3 Appendix. An approximate model for IBD sharing

To provide a simple analytical model for the relationship between total length and counts of IBD sharing, we simply consider a pair of haploid samples sharing a single diploid common ancestor at time t generations in the past and estimate the expected number and length of haplotypes shared given t (similar derivations have been made in [1–4]). We can think of the ancestry of each haploid genome as a mosaic formed by copying genomic segments from its 2^{t-1} possible ancestors. Similarly, a pair of haploid samples can be seen as a mosaic formed by copying from one ancestor for each sample. We can define paired-ancestry segments as continuous segments having no changes in ancestry in either sample. By this definition, if each sample has K chromosomes of total length L Morgans, the pair will have on average $K + 2Lt$ paired-ancestry segments.

Since each haploid sample has 2^{t-1} possible ancestors from which to inherit genetic material, a pair of samples will both inherit a paired-ancestry segment from their common ancestor with probability $\frac{1}{2^{2t-2}}$. Since the ancestor is diploid, they inherit from the same ancestral copy of the genome with probability $\frac{1}{2}$. The probability that a paired-ancestry segment is IBD in the pair is therefore $\frac{1}{2^{2t-1}}$, and the expected number of IBD segments s between the pair is:

$$s = \frac{K + 2Lt}{2^{2t-1}}. \tag{1}$$

The length of the genome shared, denoted by x, corresponds to L times the probability of having a shared ancestor at any particular locus, which is $\frac{1}{2^{2t-1}}$, giving:

$$x = \frac{L}{2^{2t-1}}. \tag{2}$$

The expected values (s, x) are shown in Fig 3 in main text as white dots for t from 1 to 5 generations, corresponding to half-siblings, first half-cousins, and so on.

Under monogamy, a similar argument can be used to obtain

$$s = \frac{K + 2Lt}{2^{2t-2}}. \tag{3}$$

and

$$x = \frac{L}{2^{2t-2}}. \tag{4}$$

Similarly, avuncular relationships in monogamy have three meioses (so $K + 3L$ segments) with a sharing probability of $\frac{1}{2}$ with one of the two shared ancestors.

$$s = \frac{K + 3L}{2} \text{ and } x = \frac{L}{2}, \tag{5}$$

and grandparent-offspring have two meioses ($K + 2L$ segments) wth a sharing probability of $\frac{1}{2}$ between the two haplotypes of the grandparent.

$$s = \frac{K + 2L}{2} \text{ and } x = \frac{L}{2}. \tag{6}$$

However, we expect that meioses occurring in the grandparent could be hidden by the statistical phasing, so that this result would be particularly sensitive to inaccurately phased data.

References

[1] A. Thomas, M. H. Skolnick, and C. M. Lewis. "Genomic mismatch scanning in pedigrees". In: *Mathematical Medicine and Biology* 11.1 (1994), pp. 1–16.

[2] C. D. Huff, D. J. Witherspoon, T. S. Simonson, J. Xing, W. S. Watkins, Y. Zhang, T. M. Tuohy, D. W. Neklason, R. W. Burt, S. L. Guthery, et al. "Maximum-likelihood estimation of recent shared ancestry (ERSA)". In: *Genome Research* 21.5 (2011), pp. 768–774.

[3] Y. Erlich, T. Shor, I. Pe'er, and S. Carmi. "Identity inference of genomic data using long-range familial searches". In: *Science (New York, N.Y.)* 362.6415 (2018), pp. 690–694.

[4] K. P. Donnelly. “The probability that related individuals share some section of genome identical by descent”. In: *Theoretical Population Biology* 23.1 (1983), pp. 34–63.
