# Supplementary material for: Accounting for long-range correlations in genome-wide simulations of large cohorts
Source: PLoS Genet. 2020 May 5;16(5):e1008619. doi: 10.1371/journal.pgen.1008619 (PMC7266353; doi:10.1371/journal.pgen.1008619)
Supplement: S4 Appendix — (PDF) [file pgen.1008619.s004.pdf]

S4 Appendix. The Genizon Biobank

The Genizon Biobank is composed of 26 cohorts for complex diseases and one general control cohort, totalling 44,981 participants. It is administered by Genome Quebec and was originally collected by Genizon BioSciences Inc. Participants were all residents of the province of Quebec [1].

We calculated IBD using genotypes from all cohorts (except Asthma and Crohn disease which were genotyped with different chips), totalling 9,961 individuals (cases and controls) including trios and duos. We removed trios and duos keeping the parents when possible, as well as individuals with less than 99% genotype calls over all SNPs. This left us with 8,435 individuals genotyped for 233,927 SNPs (keeping SNPs with at least 99% genotypes over all individuals). This filtering was done on data from each cohort individually using plink version 1.90, and they were then merged. Phasing was done using shapeit version 2.r790 and IBD was estimated using GERMLINE 1.5.1, using a minimum segment length of 5 centimorgans. Very high IBD peaks on chromosomes 1 and 9 were removed using an in-house script.

References

[1] Genome Quebec. *Genizon Biobank*. `http://www.genomequebec.com/genizon-biobank/`. Accessed January 7, 2020. (2020).
