# Supplementary material for: Accounting for long-range correlations in genome-wide simulations of large cohorts
Source: PLoS Genet. 2020 May 5;16(5):e1008619. doi: 10.1371/journal.pgen.1008619 (PMC7266353; doi:10.1371/journal.pgen.1008619)
Supplement: S1 Table — (PDF) [file pgen.1008619.s005.pdf]

**S1 Table. Relative difference in mean number of singletons, doubletons, and tripletons under the Wright-Fisher ($N_{WF}$) and Hudson ($N_H$) models.**

| **Frequency** | $\frac{N_{WF}-N_H}{N_{WF}}$ |
|---|---|
| Singletons | 0.099131 |
| Doubletons | -0.047253 |
| Tripletons | 0.010092 |

From data shown in S1 Figure. These results closely match those presented in [1, 2].

# References


[1] A. Bhaskar, A. G. Clark, and Y. S. Song. "Distortion of genealogical properties when the sample is very large." In: *Proceedings of the National Academy of Sciences of the United States of America* 111.6 (2014), pp. 2385–90.

[2] P. F. Palamara. "ARGON: fast, whole-genome simulation of the discrete time Wright-fisher process". In: *Bioinformatics* 32.19 (June 2016), pp. 3032–3034.
