# Supplementary material for: Accounting for long-range correlations in genome-wide simulations of large cohorts
Source: PLoS Genet. 2020 May 5;16(5):e1008619. doi: 10.1371/journal.pgen.1008619 (PMC7266353; doi:10.1371/journal.pgen.1008619)
Supplement: S1 Fig — A 1Mb region was simulated 100 times in 20,000 haploid lineages in a diploid population of 10,000 individuals. (PDF) [file pgen.1008619.s006.pdf]

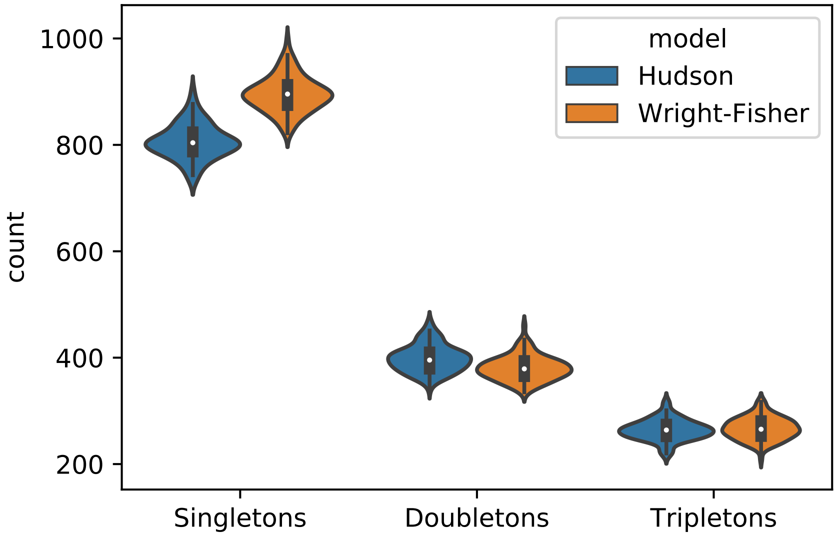

S1 Figure. Number of singletons, doubletons, and tripletons simulated under Wright-Fisher and Hudson coalescent models. A 1Mb region was simulated 100 times in 20,000 haploid lineages in a diploid population of 10,000 individuals.
