# Supplementary material for: Accounting for long-range correlations in genome-wide simulations of large cohorts
Source: PLoS Genet. 2020 May 5;16(5):e1008619. doi: 10.1371/journal.pgen.1008619 (PMC7266353; doi:10.1371/journal.pgen.1008619)
Supplement: S2 Fig — We simulated 10,000 haploid whole genomes with 22 chromosomes of realistic lengths in a population of 10,000 diploid individuals. The method for simulating multiple chromosomes is described in S1 Appendix. Similar results were shown in [21]. (PDF) [file pgen.1008619.s007.pdf]

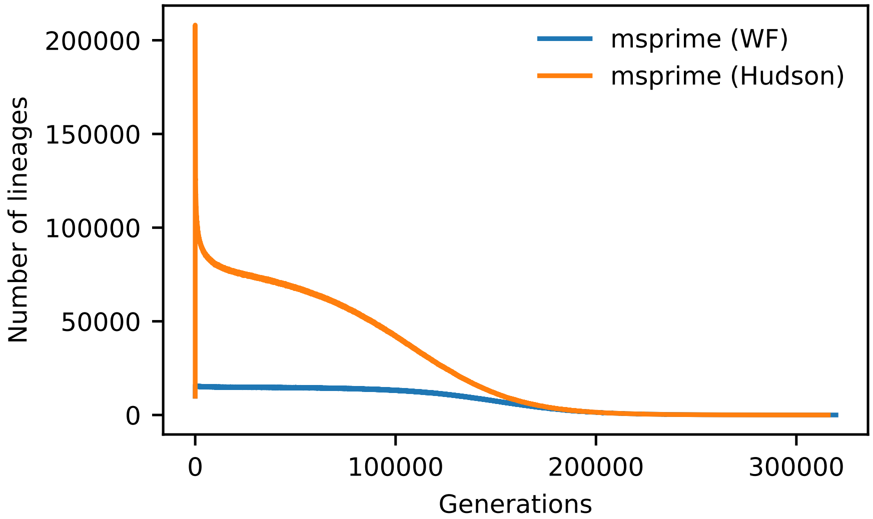

S2 Figure. Number of surviving lineages over time in coalescent and back-in-time Wright-Fisher dynamics. We simulated 10,000 haploid whole genomes with 22 chromosomes of realistic lengths in a population of 10,000 diploid individuals. The method for simulating multiple chromosomes is described in S1 Appendix. Similar results were shown in [1].

References

[1] J. L. Davies, F. Simančík, R. Lyngsø, T. Mailund, and J. Hein. "On recombination-induced multiple and simultaneous coalescent events". In: *Genetics* 177.4 (2007), pp. 2151–2160.
