# Supplementary material for: Accounting for long-range correlations in genome-wide simulations of large cohorts
Source: PLoS Genet. 2020 May 5;16(5):e1008619. doi: 10.1371/journal.pgen.1008619 (PMC7266353; doi:10.1371/journal.pgen.1008619)
Supplement: S3 Fig — 22 chromosomes of realistic lengths, simulated under Wright-Fisher model (top) and coalescent (bottom), compared to the analytical expectation under Eqs (1) and (2) in S3 Appendix. Effective population size 10,000, sample size A) 5000, B) 2500, C) 1000, D) 500. Minimum IBD segment length of 1 centimorgan. (PDF) [file pgen.1008619.s008.pdf]

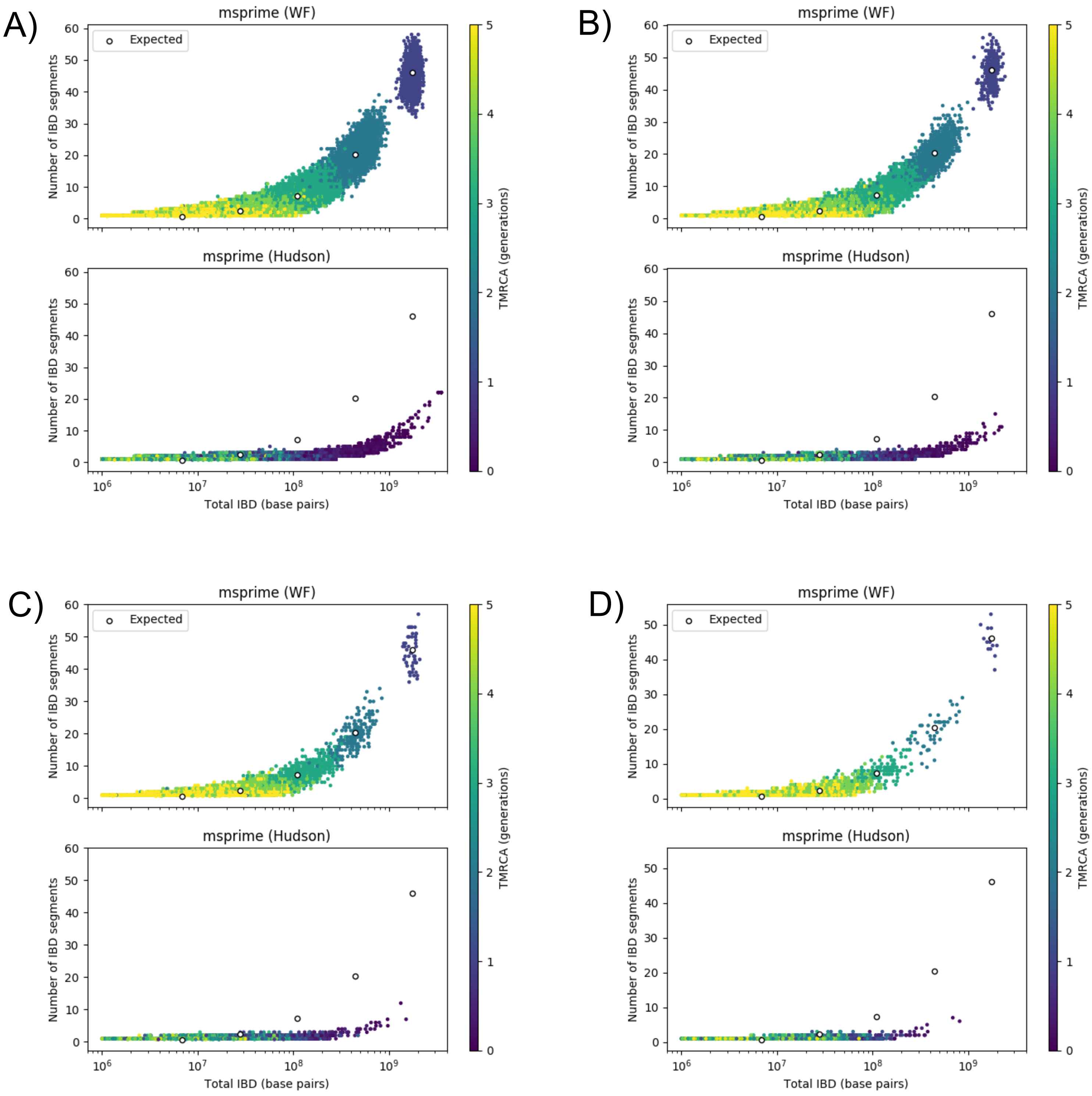

S3 Figure. Number of IBD segments between pairs of individuals versus total length of shared IBD segments. 22 chromosomes of realistic lengths, simulated under Wright-Fisher model (top) and coalescent (bottom), compared to the analytical expectation under Eqs (1) and (2) of S3 Appendix. Effective population size 10,000, sample size A) 5000, B) 2500, C) 1000, D) 500. Minimum IBD segment length of 1 centimorgan.
