# Supplementary material for: Accounting for long-range correlations in genome-wide simulations of large cohorts
Source: PLoS Genet. 2020 May 5;16(5):e1008619. doi: 10.1371/journal.pgen.1008619 (PMC7266353; doi:10.1371/journal.pgen.1008619)
Supplement: S4 Fig — 22 chromosomes of realistic lengths, simulated under Wright-Fisher model (top) and coalescent (bottom), compared to the analytical expectation under Eqs (1) and (2) in S3 Appendix. The African, European, and Asian populations had 1000 haploid samples each. (PDF) [file pgen.1008619.s009.pdf]

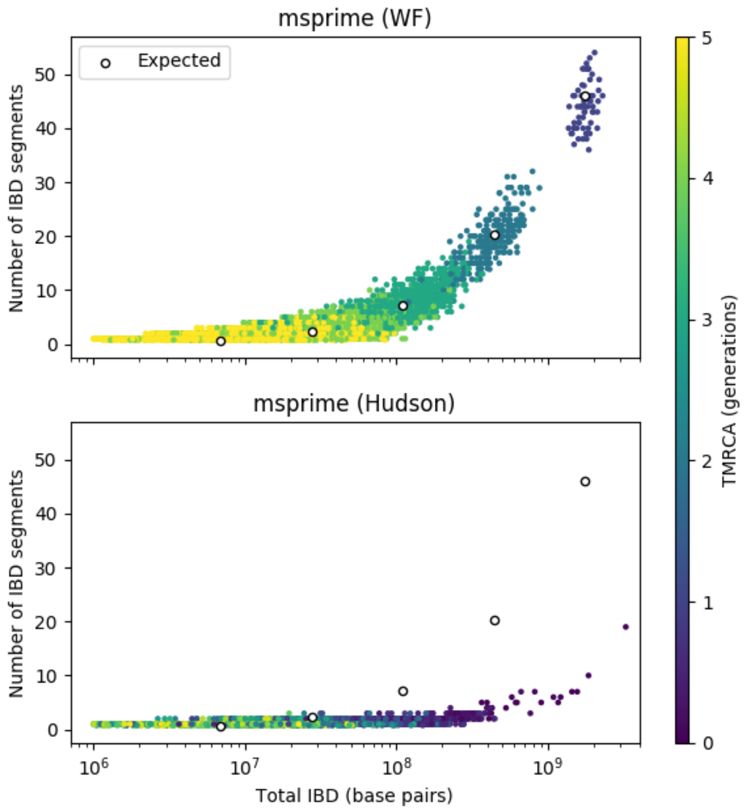

S4 Figure. Number of IBD segments between pairs of individuals versus total length of shared IBD segments, under the Gutenkunst et. al. (2009) [1] out-of-Africa model. 22 chromosomes of realistic lengths, simulated under Wright-Fisher model (top) and coalescent (bottom), compared to the analytical expectation under Eqs (1) and (2) of S3 Appendix. The African, European, and Asian populations had 1000 haploid samples each.

References

[1] R. N. Gutenkunst, R. D. Hernandez, S. H. Williamson, and C. D. Bustamante. "Inferring the joint demographic history of multiple populations from multidimensional SNP frequency data". In: *PLoS Genetics* 5.10 (2009).
