# Supplementary material for: Accounting for long-range correlations in genome-wide simulations of large cohorts
Source: PLoS Genet. 2020 May 5;16(5):e1008619. doi: 10.1371/journal.pgen.1008619 (PMC7266353; doi:10.1371/journal.pgen.1008619)
Supplement: S5 Fig — Simulations contain from 1 to 22 chromosomes of realistic lengths, using the method described in S1 Appendix, in 500 haploid samples within a diploid population of size 500. (PDF) [file pgen.1008619.s010.pdf]

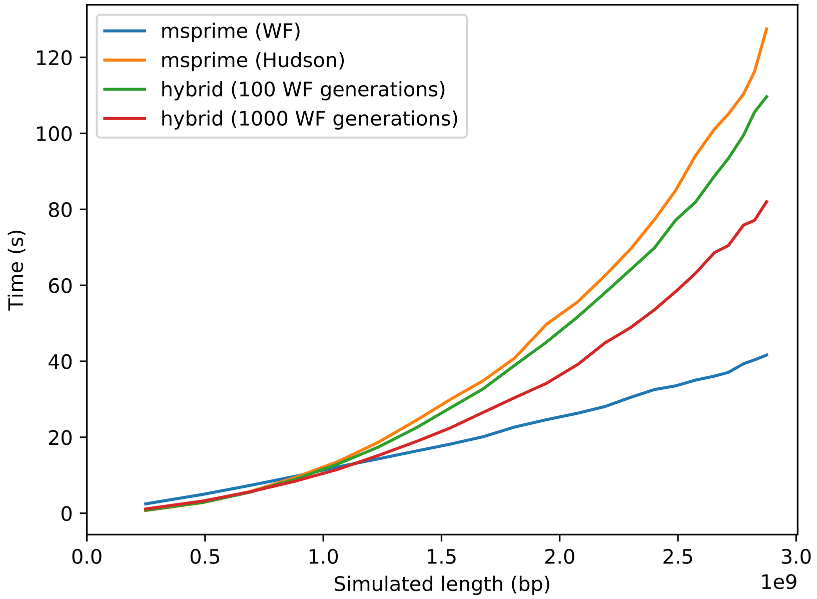

S5 Figure. Computation time of Hudson coalescent, Wright-Fisher, and hybrid models with 100 and 1000 Wright-Fisher generations before switching to the coalescent. Simulations contain from 1 to 22 chromosomes of realistic lengths, using the method described in S1 Appendix, in 500 haploid samples within a diploid population of size 500.
