# Supplementary material for: Accounting for long-range correlations in genome-wide simulations of large cohorts
Source: PLoS Genet. 2020 May 5;16(5):e1008619. doi: 10.1371/journal.pgen.1008619 (PMC7266353; doi:10.1371/journal.pgen.1008619)
Supplement: Attachment — Submitted filename: Response to reviewers.pdf [file pgen.1008619.s011.pdf]

First, we would like to thank the reviewers for their thoughtful and attentive comments on our manuscript. We're happy to have the opportunity to improve our manuscript by addressing their concerns and suggestions. Below we address each of the comments in turn, and our responses are inset.

== Reviewer #1 ==

In this manuscript the authors describe an implementation of a discrete time Wright Fisher model as part of the msprime package. The authors show how the coalescent approximation breaks down when the sample size, call it n, approaches the effective population size, Ne, and when large regions of the genome are simulated. While this result has been known for some time, the authors describe new facets to this issue and provide an implemented solution. The DTWF model that they implement compares favorably to the Hudson coalescent with respect to runtime and adequately captures features of the genealogical process that the coalescent approximation can not.

Generally I believe this to be a significant contribution, however the manuscript as written needs some substantial revision. I have point by point criticisms and suggestions that follow.

1) Generally it would be helpful to explore at what ratio of n/Ne these issues manifest. I would suggest revising the figures 2 and 3 to include a number of n/Ne ratios to show how this scaling affects the fit of the coalescent approximation.

- An excellent suggestion - we have added several n/Ne ratios to Figure 2, as well as to Supplemental Figure S3.

2) Figure 1A is very hard to follow. It certainly does not clarify what is going on. I'd suggest the authors create a new figure to describe things.

- We agree. Former figure 1A attempted to communicate too much information, but failed to do so. We have simplified Fig. 1 to show lineages and parental genomes to highlight the effects of the Wright-Fisher and Hudson recombination models.

3) Lines 87-90 in the motivation section. Is this issue purely a consequence of diploidy not being modeled? If the authors could explain the rationale a bit here it would be helpful.

- This was unclear as written, but it is a result of the limited recombination model, specifically that every recombination event creates a new, independent lineage. Lines 81 - 86 now read:
    - "This excess of ancestors is a side effect of how Hudson's coalescent algorithm models recombination. Hudson's coalescent model assumes a small region being simulated [15], and so does not account for multiple simultaneous recombinations during meiosis. The per-generation recombination rate in long genomic regions is maintained by multiple recombinations occurring at different times, with each recombination introducing a new ancestral lineage. This can lead to more than two ancestors within one generation (Fig 1)."

- The new Fig. 1 also now highlights the effects of the different recombination models.

4) Also with respect to motivation—the authors currently look at IBD tract lengths and the variance in ancestry as potential issues with the coalescent approximation. While this is great, both of those essentially are two facets of the same issue—recombination not being adequately captured by the coalescent. Can the authors look at different aspects of the data? For instance is the SFS perturbed in this regime under the coalescent?

- We agree that the IBD and ancestry issues are both consequences of the same underlying issue with the Hudson coalescent. We find it useful to highlight both issues as common analyses that can be affected by the bias, and which are resolved by the current approach.
- We also present differences in the number of singletons, doubletons, and tripletons, replicating and extending results on singletons and doubletons in Bhaskar et al. (2014). A focus on rare variants is motivated by the fact that model differences are largest in the recent past. From lines 206-210:
  - "Bhaskar et al. [13] showed that simultaneous coalescences in the Wright-Fisher model lead to more singletons and fewer doubletons than in the coalescent, which was verified in [14]. Fig S1 and Table S1 replicate these single-locus results. King et al. [17] pointed out correlation patterns among unlinked loci induced by genealogical relatedness – these results correspond to the infinite-recombination distance in Fig S5."
- We also examine long-range LD decay, which is limited by effective population size. From lines 173-177:
  - "The long-range correlations induced by genealogical relatedness can also be measured as linkage disequilibrium between distant loci. This LD is used to estimate sizes of small populations in conservation genetics [20]. Hudson's coalescent does not capture such LD patterns, whereas the Wright-Fisher extension to msprime predicts the patterns of LD expected under diploid mating (Fig S6)."

5) Line 134—typo here. No section given.

- This now points to the Performance section

6) Fig 3—unclear what the units of TMRA (shown in colors) are. Generations? Also in that figure—why is there a large gap in the data points in the top panel?

- The figure now specifies that TMRCA is measured in generations. The caption of Fig. 3 also now explains:
  - "The isolated cluster in the Wright-Fisher simulations reflects the discrete nature of possible genealogical relationships (siblings, cousins, etc.) in the Wright-Fisher model."

7) Figure 5 caption—the caption says that the sample was 1000 haploids but Ne=10000 diploids. Is this a typo? Was the actual sample size 10000 haploids?

- This has been reworded slightly as “1,000 haploid samples drawn from a diploid population of constant size 10,000” to clarify.

8) With respect to hybrid models—it would be good to show how IBD and LD are affected by the hybrid model – are these features faithfully captured by using the hybrid models?

- Since IBD is a product of recent genealogical structure, it would be unchanged under a hybrid model unless very few Wright-Fisher generations were simulated (Fig. 3 shows IBD segments inherited from common ancestors 5 generations or less in the past).
- Similarly, LD decay under the hybrid model requires very few Wright-Fisher generations to match pure Wright-Fisher simulations. Fig S5 shows that after only 2 Wright-Fisher generations the two models are indistinguishable.

9) With respect to the performance analysis—it looks like the DTWF outperforms the coalescent model starting at 1e9 bp. While this is fine, we almost never have to simulate a billion bp chromosome and instead we can simulate unlinked chromosomes as separate, one from another. The authors should probably point this out.

- There are real differences between simulating unlinked chromosomes together and simulating them separately. If done separately, a new genealogy will be generated for each simulation, which means individuals with a recent common ancestor in one simulation will likely not share the same recent ancestor in another. This can dramatically change IBD sharing among sibling pairs, for example, who can be expected to share IBD within every chromosome. Reflecting this in simulations requires that all chromosomes be simulated simultaneously. Multi-chromosome simulations are referred to in the following places:
    - Lines 92-94: “For example, samples with a recent migrant ancestor are likely to have migrant ancestry in several chromosomes, and this is not accounted for by Hudson’s coalescent.”
    - Lines 127-130: “Under the Wright-Fisher model, diploid inheritance constrains the possible gene genealogies [12] and introduces correlations in IBD sharing along long simulated regions: two samples with a recent common ancestor may be IBD at several distant regions of their genome (for example on different chromosomes).”

10) In the Supplement the authors should spend more time describing the implementation. It is very non-technical at this point. Also the authors might point the reader to the code.

- We intended this section to help users interpret the various simulation parameters - for instance whether it is possible for samples to be migrants (yes, since migration happens before breeding). Also because msprime is under active development, more technical implementation details may become out-of-date as features are added or improved. To help more technical readers however, we have added the following:
    - Pseudocode of the main simulation algorithm following line 427

   - Lines 421-423 point to up-to-date documentation: “For more technical implementation details see the documentation at https://msprime.readthedocs.io, as well as the source code at https://github.com/tskit-dev/msprime.”

11) Line 382—typo “underestimated”

- Thank you - fixed

12) Last point—the authors should show how the issue of large samples not being adequately modeled under the coalescent is realized in empirical data. For instance the authors could analyse IBD tract lengths in the UK Biobank dataset and show that the distribution observed does not square with a coalescent process. As written the paper feels more like a technical computing note than a genetics paper.

- This is an excellent suggestion, which underscores the significance of the work. We have added to Fig. 3 the distribution of IBD within a cohort of 8,435 individuals from the province of Quebec, which matches the Wright-Fisher simulations much better than the coalescent model.

== Reviewer #2 ==
This manuscript proposes a Wright-Fisher extension of msprime, a well-used coalescent simulator. Clearly this is a useful extension, but I feel that further work is needed for publication in PLoS Genetics.

1) First, it is disappointing to see only simulation results under a constant population size model. The authors should explore more realistic demographic models (e.g., previously inferred human population histories with two phases of exponential growth in the recent past) and study the accuracy of the standard coalescent model under those scenarios.

- We feel that constant population sizes are sufficient because the effects we describe are largely limited to the very recent past, where simplified demography is a reasonable approximation. We find that such simulations are able to convey the differences between the Wright-Fisher and coalescent models. The differences highlighted in Fig. 3 for example depend only on the presence of close relatives among sampled individuals, which depends only on the most recent few generations. To highlight this, we have added the following:
   - Lines 158-168: “Thus the overall relationship between IBD counts and IBD length shown on Fig 3 is a result of the discrete relatedness possibilities in diploid inheritance and does not depend on the details of the demographic history or sample sizes (see Figs S3 and S4 for simulations under different models). Of course, the number of close relatives changes with sample and population size so that the problem is more acute for large sample sizes, but Fig S3 shows clear differences between Wright-Fisher and coalescent models with Ne = 10, 000 and

500 samples. More generally, Shchur et. al. (2018) [23] calculated the expected number of p-th cousins in a sample of size K taken from a population of effective size N. In a monogamous Wright-Fisher population, when K/N = 0.2, we expect approximately 55% of samples to have a first cousin, and 95% to have a second cousin within the cohort."

- To verify our intuition, we reproduced Fig. 3 under the Gutenkunst et. al. (2009) Out-of-Africa model, simulating 1000 haploid whole genomes in each of the African, European, and Asian populations. This is shown in Figure S4, and very closely matches the results from constant-sized populations.

2) The authors have not directly demonstrated that using the WF model produces a better fit to real data. For example, it would be interesting to compare the IBD length distribution estimated from real data with simulation results from msprime (WF) and msprime (Hudson) under an inferred demographic model (e.g., inferred using the site frequency spectrum).

- This is an excellent suggestion, which underscores the significance of the work. We have added to Fig. 3 the distribution of IBD within a cohort of 8,435 individuals from the province of Quebec, which closely matches the Wright-Fisher simulations. Since the differences between the Wright-Fisher and coalescent models mostly concerns the recent past (~5 generations), we found that a simple constant-sized demographic model fit the data well.

3) Co-author Kelleher has done interesting work on simulating pedigrees. It would be natural to think of a hybrid model where a pre-specified pedigree or a probabilistic pedigree model is used for the recent past, followed by the standard coalescent in the distant past. This would be a welcome extension and could be more useful than the WF extension. After all, the WF model is rather simple and idealized, while the actual mating pattern in real populations is much more complicated. Related to this point, would it be possible to incorporate other random mating models (e.g., general Cannings exchangeable models) into msprime?

- We agree! Unfortunately this extension, while it is in our to-do list, requires extensive additional coding and testing. As noted the Wright-Fisher model is quite simple, yet implementing it within a large (and multi-language) software library while maintaining state-of-the-art performance was non-trivial. Implementing pre-specified pedigrees (along with alternate random mating models) is a natural next step, but it requires careful thought to avoid excess computational and memory overhead, as well as handling of missing records, etc. We therefore leave this for future work.

4) Since one of the main motivations for the WF extension concerns IBD sharing, it seems important to implement crossover interference. If this is not an overly difficult extension, I would strongly recommend implementing it.

- We agree, and this is indeed a planned feature for a future release. We feel however that it is more natural to examine crossover interference in the context of other factors

affecting IBD such as sex-specific recombination maps and realistic genealogies. We propose to implement and describe these finer patterns in a future publication.

5) Please explain why msprime (WF) is faster than the previous version of msprime, as shown in Figure 5. Is it because the number of lineages is bounded by the population size in "msprime (WF)", as shown in Figure 2? It would be good to discuss Figure 5 in the context of Figure 2. Related to this point, please explain why "hybrid (100 WF generations)" is slower than "msprime (Hudson)", while "hybrid (1000 WF generations)" is faster than "msprime (Hudson)". To determine the optimal switch time in the hybrid model, it seems that one should investigate the trade-off between the computational overhead for using the WF model and the reduction in the number of lineages. This suggests that the optimal switch time would depend on the demographic model. This point should be clarified. Similarly, the authors should explain why "msprime (WF)" is less efficient than "msprime (Hudson)" for shorter regions, by discussing the trade-off mentioned above.

- Your intuition is correct - the smaller number of lineages in the Wright-Fisher model is the key to the performance advantage. This was mentioned in the original manuscript starting on line 222:
    - “However, Fig 2 shows that the number of lineages in whole-genome coalescent simulations is so high that the time between events will on average be much less than a single generation. Furthermore, these lineages come at an additional memory and computational cost for the coalescent model.”
- To better explain why the Hudson model is faster for short regions, we have extended the following (last sentence new):
    - Lines 216-222: “Hudson’s coalescent algorithm avoids simulating recombination and coalescent events that do not affect genetic variation in the present sample. Whereas our Wright-Fisher implementation must iterate over all discrete generations, Hudson’s coalescent can traverse long stretches of time in a single step if there are no such events. The Hudson model is therefore more efficient than the Wright-Fisher model when the number of lineages is small, as can happen in small samples and short genomic regions, or in the distant past.”
- To clarify the relationship between hybrid switching time and demographic models, we have added:
    - Lines 229-231: “Since the optimal switching time depends on the number of extant lineages and total length of uncoalesced ancestral material, it will vary between different demographic models.”
- Clarifying why the hybrid model is not always faster than the Hudson is an important point. There is some performance overhead when switching models. In the demographic model used for performance testing (Ne = 10,000, k = 1,000), the performance advantage of 100 generations of the Wright-Fisher model was not enough to overcome the cost of switching models. The following has been added:
    - Lines 236-238: “There is a small performance cost to switching models, which explains the slightly longer runtime for the hybrid model with 100 Wright-Fisher generations versus pure coalescent simulations.”

6) My understanding is that Bhaskar et al. (PNAS 2014, 111:2385-2390) first proposed the hybrid model, but this is not clearly acknowledged in the manuscript. The first three pages of the manuscript (including the title) give the impression that the idea is being proposed here for the first time.

- It was certainly not our intention to claim to be the first to propose a hybrid simulation model. The title now reads:
    - “Accounting for long-range correlations in genome-wide simulations of large cohorts”
- Lines 70-73 now read:
    - “Using a hybrid approach with Wright-Fisher dynamics in the recent past and coalescent dynamics further back in time (as was done in [13 - Bhaskar et. al. 2014]) preserves the computational advantages of the coalescent with the long-range accuracy of the Wright-Fisher model for shorter genomic regions.”

Minor comments:

- Figure 1A: This figure is difficult to understand. Please explain it more clearly in the caption.
    - Fig 1 has now been streamlined. It is now a single figure, showing lineages and parental genomes to highlight the effects of the Wright-Fisher and Hudson recombination models.
- Figure 2 : Perhaps this should be plotted with the x-axis in log scale? Also, it would not hurt to mention that the x-axis is in "Generations (backwards in time)".
    - Helpful suggestions - scaling is now logarithmic and x-axis is now labelled “Generations (in the past)”
- Figure 3 : In the top figure, please explain why there are few IBD segments of length between ~7*10^8 and ~10^9.
    - The caption of Fig. 3 now explains that the gap in IBD segment length is a result of the discrete nature of genealogical relationships (siblings, cousins, etc.) in the Wright-Fisher model.
- Line 81-82: "We traced this phenomenon to samples having more than 2^t simulated ancestors at generation t in the past" is ambiguous. I think you meant, "We traced this phenomenon to some individuals in the sample having..."
    - This is now clarified as quoted below. Note that it was not limited to only some individuals: all individuals have an excessive number of ancestral lineages. :
        - Lines 78-80: “This is because individuals had too many simulated ancestors: whereas diploid individuals carry at most 2^t ancestors at generation t in the past, coalescent simulations allow for many more ancestors.”
- Line 95-96: It would help the reader to explain here why recent events in migration models induce long-range correlations along the genome.
    - Lines 92-94 now read: “For example, samples with a recent migrant ancestor are likely to have migrant ancestry in several chromosomes, and this is not accounted for by Hudson's coalescent.”

- Lines 101-105: Bhaskar et al. (2014) compared the WF and the coalescent models with respect to the number of lineages at a single site. It would be good to discuss this result in relation to your result.

- A good suggestion - lines 206-208 now read:
    - “Bhaskar et al. [13] showed that simultaneous coalescences in the Wright-Fisher model lead to more singletons and fewer doubletons than in the coalescent, which was verified in [14]. Fig S1 and Table S1 replicate these single-locus results.”

- There are blank references to sections throughout the manuscript. For example, Figure 2 caption ends with "described in Section ."

- Thank you - section references have been fixed.

- Line 140: Replace "closely related samples" with "closely related individuals".

- As suggested, lines 152-153 now read:
    - “By contrast, the coalescent model exhibits far too few IBD segments for closely related individuals and poor clustering by TMRCA.”
