# Supplementary material for: Accounting for long-range correlations in genome-wide simulations of large cohorts
Source: PLoS Genet. 2020 May 5;16(5):e1008619. doi: 10.1371/journal.pgen.1008619 (PMC7266353; doi:10.1371/journal.pgen.1008619)
Supplement: Attachment — Submitted filename: Response to reviewers #2.pdf [file pgen.1008619.s012.pdf]

We would again like to thank the reviewers for their thoughtful comments, which have greatly improved our manuscript. We address the remaining comments below.

Reviewer #1: I'm pleased with the edits made to this revision. This is an excellent contribution.

- Thank you for your comments!

Reviewer #2: Overall the authors have done a good job of revising the paper and I am generally satisfied with all the changes. One exception is their response to my first major comment regarding the effect of demography on the distribution of pairwise IBD length. The authors have done simulation using the Out-of-Africa model from Gutenkunst et al. (2009), but my understanding is that in that model the present effective population sizes of YRI, CEU, and CHB are 7300, 29524, and 53403, respectively. What would happen if the present effective population size were much larger, say 1 million or 10 million, while the sample size is held at 1000? The authors claim, "the overall relationship between IBD counts and IBD length ... does not depend on the details of the demographic history or sample sizes." To me, this seems like a strong claim which warrants more rigorous justification, as it might send an incorrect message to the reader. To what extent does it not depend on the demographic model? Could you be more quantitative?

- This has been clarified to highlight the points you raise. While the distribution of IBD segments among related individuals is primarily a function of their degree of relatedness, the number of closely-related individuals is certainly a function of demography. Lines 162-171 now read:

"The distribution of long IBD segments between related individuals is primarily determined by their degree of recent relatedness. For example, even though the population history and sampling process affects the *number* of sampled first cousins, the recent IBD relatedness *among* first cousins in large outbred populations is relatively independent of history and sampling: this is why the simulated and empirical distributions observed on Fig 3 are in good agreement despite differences in population sizes, and why the theoretical predictions that describe both are independent of the population demography. Because the number of close relatives changes with sampling and population size, the discrepancy between coalescent and Wright-Fisher models is more acute for large sample sizes (see Figs S3 and S4 for simulations under different models)."
